# Anatomic and Functional Outcomes of Vitrectomy for Advanced Retinopathy of Prematurity: A Systematic Review

**DOI:** 10.18502/jovr.v19i2.14027

**Published:** 2024-06-21

**Authors:** Anthea Casey, Raihanny Andrea Zahra, Julie Dewi Barliana

**Affiliations:** ^1^Department of Ophthalmology, Faculty of Medicine, Universitas Indonesia, Dr. Cipto Mangunkusumo Hospital, Jakarta, Indonesia; ^2^Pediatric Ophthalmology Division, Department of Ophthalmology, Faculty of Medicine, Universitas Indonesia, Dr. Cipto Mangunkusumo Hospital, Jakarta, Indonesia

**Keywords:** Infant; Newborn; Retinopathy of Prematurity; Treatment Outcome; Vitrectomy

## Abstract

The success of vitrectomy in the advanced stages of retinopathy of prematurity (ROP) is defined not only by anatomical results, but also by functional outcomes. Studies have indicated that vitrectomy produces better outcomes when performed at an earlier stage (stage 4 vs. stage 5 ROP). This study reviewed the outcomes of vitrectomy in advanced stages of ROP and the associated factors. PubMed, ScienceDirect, Cochrane, Wiley, and WorldCat databases were systematically searched for articles published in the last 10 years. Studies involving participants with stages 4 and 5 ROP who underwent vitrectomy were included. The final search was performed on March 24, 2023. Risk of bias was assessed using the National Institutes of Health Quality Assessment Tool.The results were presented in accordance with the Preferred Reporting Items for Systematic Reviews and Meta-Analyses 2020 guidelines. Ten studies were included in the review. A total of 1179 eyes underwent vitrectomy (72% lens-sparing vitrectomy [LSV] and 28% lensectomy-vitrectomy [LV]). LSV was mainly performed in stage 4 ROP and LV in stage 5 ROP. Anatomical and functional successes were more significant in stages 4A and 4B than in stage 5. Factors that improved prognosis included no plus diseases, stage 4, prior treatments such as laser or intravitreal anti-vascular endothelial growth factor injection, and sparing the lens intraoperatively. Vitrectomy resulted in better outcomes in patients with stage 4 ROP. Early detection and a strict screening protocol are needed to prevent ROP progression into stage 5.

##  INTRODUCTION

Advances in neonatal intensive care units (NICU) have led to a significantly increased life expectancy of premature infants. As the life expectancy increases, complications and developmental disorders, including retinopathy of prematurity (ROP), may develop.^[1]^ Unfortunately, a proper ROP screening protocol still does not reach all premature babies in need.^[2,3]^ According to the Early Treatment for Retinopathy of Prematurity (ETROP) study, despite careful screening and treatment, 12% of eyes progress to stages 4–5 ROP and require surgery.^[4]^

Surgical interventions include scleral buckling and vitrectomy with or without lensectomy.^[5]^ However, ROP surgery in Indonesia and other middle-income countries is only performed in a few tertiary care centers. Late detection of retinal detachment; lack of trained pediatric ophthalmologists, pediatric anesthesiologists, postoperative neonatal support, and vision rehabilitation and supportive therapy; and poor follow-up all contribute to this problem.^[2]^ In addition, the visual outcome is generally poor despite successful anatomical retinal reattachment. Previous studies have revealed the benefits of surgical management for stage 4 ROP.^[6,7,8,9]^ However, the effectiveness of surgery for stage 5 ROP remains controversial, and existing literature discussing the outcomes of surgery for stage 5 ROP is still limited. This study aimed to review the outcomes of vitrectomy in advanced stages of ROP and the associated factors.

##  METHODS

This study was registered in PROSPERO (CRD42023411204) and adhered to the Preferred Reporting Items for Systematic Reviews and Meta-Analyses (PRISMA).^[10]^

### Eligibility Criteria

Studies involving a population of patients with stages 4 and 5 ROP who underwent vitrectomy with or without additional treatment were included. The evaluated outcomes were anatomical and/or functional success after vitrectomy. Prospective and retrospective human studies were included. Case reports or case series with < 10 participants, editorials, expert opinions, and studies with a follow-up duration of < 6 months were excluded.

### Search Strategy

Databases such as PubMed, ScienceDirect, Cochrane, Wiley, and WorldCat were systematically searched for relevant studies reporting outcomes following vitrectomy in patients with advanced ROP. The search filters included articles written in English, published over the last 10 years. The final search was performed on March 24, 2023.

### Selection Process

**Figure 1:**
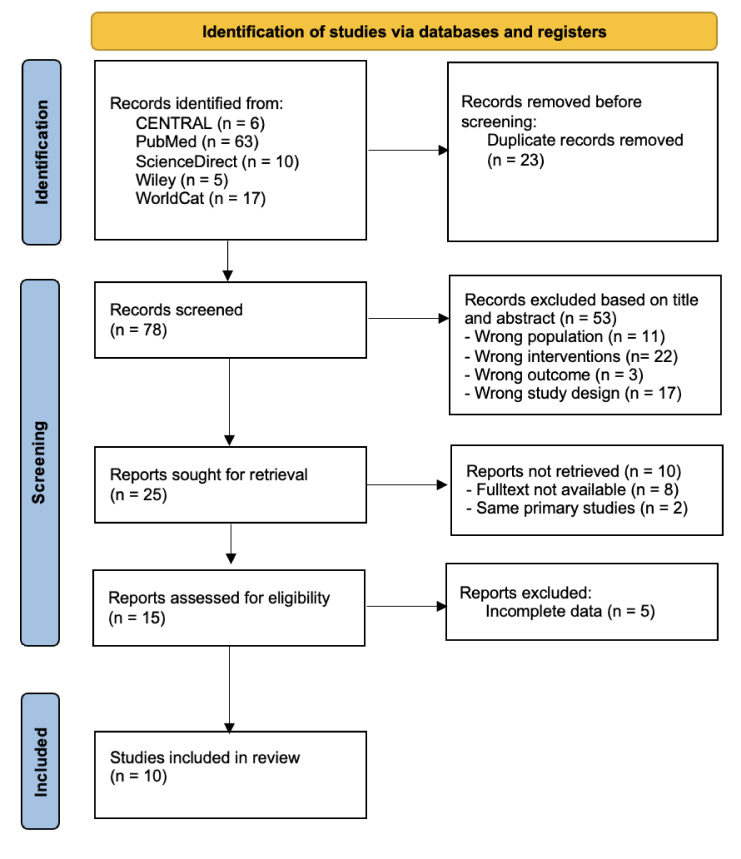
Study selection process.

All studies from each database were imported into the Rayyan Intelligent Systematic Review.^[11]^ Duplicates were removed using automated tools. All authors independently screened the article titles and abstracts for inclusion and exclusion. Disagreements were resolved through discussions. The study selection process is presented in the flow diagram recommended in the PRISMA 2020 statement [Figure 1].

### Data Extraction

Two authors (AC and RAZ) collaborated to extract the data. Gestational age (GA), birth weight (BW), postmenstrual age (PMA) at the time of surgery, and follow-up duration are presented as means ± standard deviations. The type of surgery, secondary operations, complications, and anatomical and functional success rates are presented as percentages. Factors associated with prognosis are listed as odds ratios, if available. JDB reviewed all the extracted data and checked for any missed variables.

**Table 1:** Results of the quality assessment of included studies

**Major components**	**Rajan^[1]^ (2021)**	**Özdek^[13]^ (2021)**	**Özsaygili^[14]^ (2019)**	**Gusson^[15]^ (2019)**	**Chandra^[16]^ (2019)**	**Sen^[17]^ (2019)**	**Karacorlu^[18]^ (2017)**	**Nudleman^[19]^ (2015)**	**Shah^[20]^ (2015)**	**Gadkari^[21]^ (2015)**
1. Was the study question or objective clearly stated?	Yes	Yes	Yes	Yes	Yes	Yes	Yes	Yes	Yes	Yes
2. Was the study population clearly and fully described, including a case definition?	Yes	Yes	Yes	Yes	Yes	Yes	Yes	Yes	Yes	Yes
3. Were the cases consecutive?	Yes	Not reported	Yes	Yes	Not reported	Not reported	Not reported	Not reported	Not reported	Yes
4. Were the subjects comparable?	Yes	No	No	No	No	No	No	No	No	No
5. Was the intervention clearly described?	Yes	Yes	Yes	Yes	Yes	Yes	Yes	Yes	Yes	Yes
6. Were the outcome measures clearly defined, valid, reliable, and implemented consistently across all study participants?	Yes	Yes	Yes	Yes	Yes	Yes	Yes	Yes	Yes	Yes
7. Was the length of follow-up adequate?	Yes	Yes	Yes	Yes	Yes	Yes	Yes	Yes	Yes	Yes
8. Were the statistical methods well-described?	Not reported	Yes	Yes	Yes	Yes	Yes	Not reported	Yes	Not reported	Yes
9. Were the results well-described?	Yes	Yes	Yes	Yes	Yes	Yes	Yes	Yes	Yes	Yes
**Overall**	**Good**	**Good**	**Good**	**Good**	**Good**	**Good**	**Good**	**Good**	**Good**	**Good**
	
	
Rajan et al^[1]^ (2021)	India (2016–2018)	21	St 5: 1185 ± 222.4 (990–1700)	St 5: 29.9 ± 2.0 (26–33)	St 5: 44.6 ± 9.8 (34–71)	St 5: 33%	St 5: 66.7%	6.33 ± 2.2 months (range: 3–12 months)	N/A
Özdek et al^[13]^ (2021)	Turkey (2010–2019)	70	Overall: 1284.7 ± 463.2 (670–2500)St 4A: 1306.5 ± 467.9 St 4B: 1330 ± 483.8 St 5: 981.7 ± 268.9	Overall: 28.6 ± 2.9 (23–35)St 4A: 28.7 ± 3.1 St 4B: 29.1 ± 2.8 St 5: 26.8 ± 1.3	Overall: 41.4 ± 4.9 (33–58)St 4A: 41.2 ± 4.6 St 4B: 43.2 ± 5.8 St 5: 37.5 ± 2.7	St 4A: 89%St 4B: 61%St 5: 83%	St 4A: 11%St 4B: 39%St 5: 17%	2.3 ± 1.7 years (range: 0.3–7 years)	22.9%*
Özsaygili et al^[14]^ (2019)	Turkey (2011–2016)	121	Overall: 1203.9 ± 434.4 (600–2580)St 4A: 1244 ± 442 St 4B: 1139 ± 418.4	Overall: 28.5 ± 2.7 (23–35)St 4A: 28.5 ± 2.8 St 4B: 28.2 ± 2.6	Overall: 43.8 ± 7.5 St 4A: 41.7 ± 5.4 St 4B: 47.1 ± 9.0	St 4A: 85.1%St 4B: 53.2%	St 4A: 14.9%St 4B: 46.8%	2 ± 0.7 years (range: 1–3.2 years)	17.4%
Gusson et al^[15]^ (2019)	Italy (1999–2013)	70	Overall: 725 (540–1215)	Overall: 26 (23–29)	Overall: 44 (20–72)	St 4B: 26.1%St 5: 10.6%	St 4B: 73.9%St 5: 89.4%	8.5 years (2–16 years)	40%
Chandra et al^[16]^ (2019)	India (2015–2017)	60	Overall: 1214.5 ± 329.7 (750–1800)	Overall: 28.4 ± 2.0 (26–32)	Overall: 40.8 ± 2.2 (37–45)	St 4A: 96.2%St 4B: 87.5%	St 4A: 3.8%St 4B: 12.5%	11.3 months (5–17 months)	1.7%
Sen et al^[17]^ (2019)	India (2012–2015)	202	Overall: 1327 ± 440 (600–2800)	Overall: 29.5 ± 2.8 (24–38)	Overall: 51.7 (30–104)	St 4A: 79%St 4B: 49%St 5: 4%	St 4A: 21%St 4B: 51%St 5: 96%	8.1 months (1.5–39 months)	N/A
Karacorlu et al^[18]^ (2017)	Turkey (1996–2010)	88	Overall: 1212 ± 355 St 4A: 1202 ± 331 St 4B: 1180 ± 412 St 5: 1257 ± 293	Overall: 28.4 ± 2.3 St 4A: 28.0 ± 1.7 St 4B: 28.5 ± 2.5 St 5: 28.7 ± 2.3	Overall: 43.1 ± 6.2 St 4A: 41.6 ± 6.3 St 4B: 43.1 ± 5.6 St 5: 44.0 ± 6.8	St 4A: 79%St 4B: 63%St 5: 0%	St 4A: 21%St 4B: 37%St 5: 100%	6.9 ± 1.9 years (up to 5 years)	8% †
Nudleman et al^[19]^ (2015)	USA (1992–2013)	496	Overall: 790.3 (350–1758)St 4A: 770.3 St 4B: 735.5 St 5: 778.2	Overall: 25.5 (20–34)St 4A: 25.5 St 4B: 25.2 St 5: 25.3	N/A	St 4A: 100%St 4B: 100%St 5: 100%	N/A	4.3 years (range: 0.002–20.1 years)	19.8%
Shah et al^[20]^ (2015)	India (2012–2014)	20	Overall: 1265 (890–1700)St 4A: 1119 St 4B: 1443	Overall: 29.8 (28–32)St 4A: 29.5 St 4B: 30.2	Overall: 38.5 (37–42)St 4A: 38.8 St 4B: 38.6	St 4A: 100%St 4B: 100%	N/A	8.7 months (range: 4–17 months)	N/A
Gadkari et al^[21]^ (2015)	India (from January 2009)	31	Overall: 1166.7 (700–1800)St 4B: 1189.5 ± 311St 5: 1076.7 ± 224.7	Overall: 30.1 (27–32)St 4B: 29.9 ± 1.7 St 5: 30.2 ± 2	St 4B: 49.8 ± 13.5 (36–76)St 5: 72.7 ± 16.6 (42–92)	St 4B: 55%St 5: 9%	St 4B: 45%St 5: 91%	6 months minimum	N/A
	
	
Add., additional; BW, birth weight; GA, gestational age; LSV, lens-sparing vitrectomy; LV, lensectomy–vitrectomy; PMA, post-menstrual age; St, stage ${\;}^{*}$ A total of 16/70 eyes (22.9%) required additional surgery, comprising 19.4% stage 4A, 22.2% stage 4B, and 50% stage 5 eyes. ${\;}^{†}$ A total of 7/88 eyes (8%) required a second surgery, comprising 21.1% stage 4A, 5.3% stage 4B, and 3.2% stage 5 eyes.									

**Table 2:** Vitrectomy outcomes in advanced stages of ROP


**Study**	**Anatomical success**	**Functional success**	**Factors associating prognosis**		**Complications**
		**Better**	**Worsen**	
Rajan et al^[1]^ (2021)	St 5: 19%	St 5: 23.8%	–	Closed – closed preoperative RD configuration	Intraoperative bleeding (9.5%)
Özdek et al^[13]^ (2021)	St 4A: 95.7%St 4B: 83.3%St 5: 50%	N/A	–	Stage 5	Vitreous hemorrhage (11.4%), strabismus (18.6%), nystagmus (20%), glaucoma (10%)
Özsaygili et al^[14]^ (2019)	St 4A: 91.3%St 4B: 65.1%	St 4A: 87.4%St 4B: 61.6%	Stage 4, preoperative treatments, LSV, PHD	Plus diseases, postoperative vitreous hemorrhage, retinal break	Vitreous hemorrhage (10.7%), strabismus (30.6%), nystagmus (13.2%)
Gusson et al^[15]^ (2019)	St 4B: 82.6%St 5: 46.8%	St 4B: 47.8%St 5: 17%	Stage 4, LSV	–	Glaucoma (14.3%), intraocular hemorrhage (8.6%), corneal opacity (4.3%), and phthisis (7.1%)
Chandra et al^[16]^ (2019)	St 4A: 71.2%St 4B: 37.5%	N/A	Stage 4	Annular TRDTRD extent 7.9 ± 3.4 clock hours	Vitreous hemorrhage (6.7%), glaucoma (1.7%)
Sen et al^[17]^ (2019)	St 4A: 74%St 4B: 74%St 5: 33%	N/A	Stage 4 (OR 5.8), preoperative treatments (OR 2.3), LSV (OR 7), 25G MIVS (OR 1.7)	Retinal break (OR 0.21)	Intraoperative break (19%), vitreous hemorrhage (28%), increased IOP (12.7%), cataract (2.4%)
Karacorlu et al^[18]^ (2017)	St 4A: 89%St 4B: 63%St 5: 42%	St 4A: 63.2%St 4B: 57.9%St 5: 35.5%	Stage 4, preoperative treatments	–	Strabismus (34%), high myopia (21.6%), corneal opacity (14.8%), glaucoma (13.6%), phthisis (9.1%), cataract (3.4%)
Nudleman et al^[10]^ (2015)	St 4A: 82.1%St 4B: 69.5%St 5: 42.6%	N/A	Stage 4	Lens opacification (26.6%)
Shah et al^[20]^ (2015)	St 4A: 100%St 4B: 89%	N/A	N/A	N/A	High myopia (45%), glaucoma (15%)
Gadkari et al^[21]^ (2015)	St 4B: 90%St 5: 45.5%	St 4B: 60%St 5: 9%	Stage 4	–	N/A
	
	
IOP, intraocular pressure; LSV, lens-sparing vitrectomy; MIVS, microincision vitrectomy; N/A, not applicable; OR, odds ratio; PHD, posterior hyaloid detachment; RD, retinal detachment; TRD, tractional retinal detachment					

### Risk of Bias Assessment

The risk of bias was assessed using the National Institutes of Health (NIH) Quality Assessment Toolfor Case-Series Studies (interventional). This tool comprised nine items, each assessed as “Yes,” “No,” or “Cannot Determine/Not Applicable/Not Reported”. The overall quality rating for each study was then concluded as “Good,” “Fair,” or “Poor.”^[12]^ Each author independently assessed the risk of bias. Discrepancies were resolved through discussion.

##  RESULTS

In total, 101 studies were identified from the five databases (63 from PubMed, 6 from Cochrane, 10 from ScienceDirect, 5 from Wiley, and 17 from WorldCat). After screening the titles and abstracts, 25 studies were selected for full-text retrieval. Ten studies (one prospective and nine retrospectives) involving 782 patients (1179 eyes) were eligible.^[1,13,14,15,16,17,18,19,20,21]^ The quality of all studies was good according to the NIH quality assessment tool [Table 1]. These studies were conducted in different parts of the world: five were conducted in India,^[1,16,17,20,21]^ three in Turkey,^[13,14,18]^ one in Italy,^[15]^ and one in the United States of America (USA).^[19]^ Most of the studies were case series; thus, the level of evidence in these studies was 4. The characteristics of these studies are summarized in Table 2**.**

Only studies with ≥ 10 participants were included in the analysis. The mean GA and mean BW were lower in Western countries than those in Asian countries.^[15,19]^ Smaller GA and BW were not associated with a more severe disease.^[18,19,20,21]^ However, two other studies have stated otherwise.^[13,14]^ Özsaygili et al have reported that the prevalence of stage 4B was significantly higher than that of stage 4A (63.8% vs 43.7%, *P* = 0.037) in patients with a GA of ≤ 28 weeks and in those with a BW of < 1000 gr (51.1% vs 35.1%, *P* = 0.049). However, plus diseases were significantly higher in stage 4A eyes than in stage 4B eyes (66.2% vs 48.9%, *P* = 0.045).^[14]^ Severe disease was defined as those with a more advanced stage and the presence of a plus disease. As demonstrated in Table 2, lower GA and lower BW was not always present in stage 5 ROP compared to stages 4A and 4B ROP. Several studies reporting the presence of a plus disease indicated a higher prevalence in stage 4A ROP than in stage 4B ROP. Sen et al reported that the percentages of a plus disease were 52% and 25.5% for stages 4A and 4B ROP, respectively.^[17]^ Similarly, Karacorlu et al reported that the percentages of plus disease were 89%, 82%, and 16% in stages 4A, 4B, and 5 ROP, respectively.^[18]^ Preoperative treatments included laser photocoagulation, intravitreal anti-vascular endothelial growth factor (VEGF) injection, or both. Most of the included studies have reported laser photocoagulation as the most commonly performed preoperative treatment for all ROP stages.^[1][13,14][15][16][18,19,20,21]^ Only one study by Sen et al have indicated that a combination of laser and anti-VEGF therapy was the most commonly performed preoperative treatment.^[17]^

As demonstrated in Table 2, lens-sparing vitrectomy (LSV) was more commonly performed in stage 4 ROP, whereas lensectomy–vitrectomy (LV) was more commonly performed in stage 5 ROP. The mean percentages of the LSV performed at stages 4A, 4B, and 5 ROP were 89.8%, 66.1%, and 34.2%, respectively. Meanwhile, the mean LV percentages in stages 4A, 4B, and 5 ROP were 28.7%, 43.6%, and 76.7%, respectively. Additional surgeries were primarily required because of postoperative vitreous hemorrhage. The mean interval between the first and second surgeries was 11.6 ± 14.9 weeks in a study by Özdeket al and 9 weeks in a study by Karacorlu et al.^[13,18]^ Özsaygili et al reported that the average duration for the second surgical procedure was 6.4 weeks in cases of postoperative hemorrhage and 43.5 weeks for staged surgeries aimed at achieving more retinal reattachment areas in partial retinal detachments.^[14]^

The anatomical and functional outcomes after vitrectomy are presented in Table 3. Anatomical success was defined as complete retinal reattachment with an undistorted or minimally distorted macula for stage 4A, complete retinal attachment or partial residual peripheral retinal detachment not involving the macular region for stage 4B, or attachment of any part of the posterior pole for stage 5.^[13,14,18]^ Anatomical success rate was the highest in stage 4A (71.2–100%), followed by stage 4B (37.5–90%), and stage 5 (19–50%). Functional success was also the highest in stage 4A (63.2–87.4%), followed by stage 4B (47.8–61.6%), and stage 5 (9–35.5%). None of these studies explicitly mentioned the age of patients at the time of functional assessment. However, this can be estimated from the PMA at the time of surgery and the mean follow-up duration [Table 2]. The definitions of functional success have slight differences. Most studies defined functional success as the presence of light perception, “fix and follow” visual acuity, or central–steady–maintained (CSM) fixation.^[1,14,15]^By contrast, Karacorlu et al reported that “fix and follow” and CSM fixation were not considered functional success.^[18]^ Although the definition of functional success varied across studies, the success rates provided in Table 3 were based on the respective definitions of success provided by each study.

Several factors contribute to a better prognosis, including stage 4 ROP, preoperative treatment, LSV, and induction of posterior hyaloid detachment.^[14,15,16,17,18,19,21]^ One study that compared 23G and 25G microincision vitrectomy surgery (MIVS) revealed that surgeries with 25G MIVS produced better outcomes.^[17]^ Meanwhile, the anteriorly closed-posteriorly closed type retinal detachment configuration,^[1]^ annular type of tractional retinal detachment,^[15]^ presence of retinal break,^[14,17]^ and postoperative vitreous hemorrhage^[14]^ contributed to poor prognosis. The most common complications after vitrectomy in patients with ROP include vitreous hemorrhage,^[13,14][15][16][17]^ glaucoma,^[13,15,16,17,18,20]^ strabismus,^[13,14,18]^ nystagmus,^[13,14]^ high myopia,^[18,20]^ and cataract.^[17,18,19]^

##  DISCUSSION

Vitrectomy has emerged as the preferred treatment for infants with advanced ROP who experience retinal detachment. In contrast to scleral buckling, vitrectomy offers several advantages, including its direct impact on tractions, the removal of various growth factors and cytokines in the vitreous that may contribute to failure, high success rates with a single surgery, absence of induced myopia, and no requirement for secondary surgical intervention.^[14]^

The ideal timing for vitrectomy should be planned carefully; that is, when vascular activity decreases and retinal detachment begins.^[13]^ Early surgery was defined as surgery performed ≤ 52 weeks PMA.^[21]^ Our review of the included studies demonstrated that surgeries were generally conducted early considering the overall PMA at the time of surgery. However, the appropriate timing for surgery in patients with stage 5 ROP remains debatable. In the study by Rajan et al, the median age at surgery was 42 weeks.^[1]^ Additionally, Sen et al reported that infants with stage 5 ROP who received surgery between 3 and 9 months of age exhibited better anatomical outcomes than those who received surgery at < 3 months of age. This signified that the status of infants' eyes with stage 5 ROP was so seriously severe at presentation that even when they were operated on early, the prognosis was poor.^[17]^

Two studies in our review, conducted in Italy and the USA, have indicated that infants with advanced ROP had a mean GA of ≤ 28 weeks and a mean BW of < 1000 gr.^[15,19]^ In contrast, other studies conducted in India and Turkey revealed a higher mean GA and BW in infants with advanced ROP. These findings are consistent with those of previous studies that reported a higher incidence of ROP among infants with higher GA and BW in Asia.^[2,21]^ However, in this study, GA and BW did not significantly affect outcomes following vitrectomy. Gadkari et al have also reported that early surgery ( ≤ 52 weeks) did not influence anatomical and functional successes (P = 0.65 and P = 0.17, respectively).^[21]^

Two types of vitrectomy were performed in the included studies: LSV and LV. LSV was considered in each case when it was possible. LSV has certain benefits over primary scleral buckle surgery and vitrectomy in combination with lensectomy. Unlike scleral buckles, LSV does not induce significant anisometropia and eliminates the need for an additional surgical procedure to remove the buckle as the eye grows. Preserving an infant's natural lens offers the advantages of supporting vision development and reducing the risk of glaucoma. Nevertheless, some situations necessitate extracting the lens to release the tractional components anteriorly.^[22]^

Lensectomy is not preferred to avoid the condition of aphakia, which can hinder the visual development of infants during a critical periods.^[13,14]^ Additionally, the decision to opt for lensectomy has significant long-term consequences, particularly regarding the elevated risk of aphakic glaucoma throughout the lifetime.^[15]^ Nudleman et al observed that the likelihood of developing glaucoma was 2.76 times higher in eyes that underwent lensectomy than in those that did not.^[19]^ Nevertheless, lensectomy was performed when extensive anterior traction extended up to the posterior lens capsule or tractions extended to the far periphery.^[13,14]^

The higher success rate observed in eyes with LSV is most likely attributable to the fact that LSV surgery was performed in less severe cases, predominantly in stage 4A eyes lacking peripheral retinal traction extending behind the lens. This finding aligns with that of our study, in which LSV was primarily performed in stage 4A cases, whereas LV was reserved for stage 5 cases.

Anatomical and functional outcomes were better at an earlier stage (stage 4 ROP), which was evident and statistically proven.^[1][13,14][15][16][17,18,19][21]^ However, the long-term outcomes reported by the ETROP Group were comparatively poor, with only 30% of the eyes that underwent vitrectomy for retinal detachment maintaining macular attachment after six years of follow-up. Long-term follow-up in the ETROP study compared to that in previous studies may have contributed to the observed discrepancies, as long-term examinations of infants with stages 4 and 5 ROP following surgery can be challenging.^[22]^

Of the 10 included studies, only 5 assessed functional outcomes.^[1,14,15][18][21]^ Assessing functional outcomes has been proven to be challenging owing to variations in visual examination and vision rehabilitation approaches. Visual acuity was assessed considering the infant's age using a suitable refractive correction. Consequently, we concur with Özsaygili's report, which defined functional success based on the age at which visual acuity assessment was conducted. These included criteria such as achieving CSM fixation within the first year of age, form vision (fixation and following small objects) between 1 and 2 years of age, and ambulatory vision (Snellen visual acuity of 20/2000 or logMAR 1.80) or better in patients aged > 2 years.^[14]^ Other studies have considered functional success as visual acuity better than light perception.^[1,15,21]^ Gusson et al graded visual function as "no light perception," "light perception," "form vision," "low vision cards" (Teller cards), and "better than low vision cards" (Snellen chart).^[15]^ Karacorlu et al measured visual acuity measurements using the Tumbling E or Early Treatment Diabetic Retinopathy Study (ETDRS) charts.^[18]^ The Snellen optotype is often considered the gold standard for determining resolvable visual acuity.^[23]^ This statement is likely the basis for the functional success in Karacorlu's study, defined by an approximation of Snellen's visual acuity equivalent with CSM and fixation following exclusion.

Visual evoked potentials were used when visual acuity testing could not be performed because of developmental limitations, neurologic abnormalities, or uncooperative patients.^[15]^ Orthoptic assessments and low-vision rehabilitation were performed to optimize visual development. Contact lenses or spectacles were prescribed to correct aphakia and refractive errors.^[15]^ Ametropic and anisometropic amblyopia were managed by correction with contact lenses or spectacles, and subsequent eye patching.^[18]^ Despite anatomical success, the lack of visual function can be attributed to non-ocular visual pathway damage related to complications associated with extreme prematurity. Additionally, the impact of retinal detachment and subsequent reattachment on the developing photoreceptor–retinal pigment epithelial complex may contribute to limited visual function.^[1,15]^

The main limitation of this study was its retrospective nature. The lack of uniformity (staging, preoperative treatments, timing of surgery, and presence of diseases) made comparisons between the series difficult. Prospective studies with standardized evaluations of anatomical and functional outcomes are required to evaluate the long-term outcomes of vitrectomy in a larger cohort of infants with ROP.

##  SUMMARY

The anatomical and functional success rates following vitreoretinal surgery were encouraging for stage 4 ROP and were poor for stage 5 ROP. Retinal reattachment resulted in better visual outcomes than the untreated natural history of advanced ROP-related retinal detachment. ROP treatment should be conducted with the aim of avoiding advanced ROP, particularly stage 5, by screening high-risk infants and doing follow-ups accordingly.
